# Armillifer armillatus infestation in Human; public health scenario of a snake parasite: a report of three cases

**DOI:** 10.11604/pamj.2016.25.45.10282

**Published:** 2016-09-29

**Authors:** Joshua Oluwafemi Aiyekomogbon, Clement Adebajo Meseko, Olugbenga Olusola Abiodun

**Affiliations:** 1 Department of Radiology, University of Abuja and Federal Medical Centre, Jabi-Airport Road, Abuja, Nigeria; 2 Regional Centre for Avian Influenza (AI) and Transboudary Animal Diseases, National Veternary Research Institute, Vom, Jos, Plateau State, Nigeria; 3 Department of Medicine, Federal Medical Centre, Jabi-airport Road, Abuja Nigeria

**Keywords:** Armillifer armillatus, snake, reptiles, computed tomography, chest radiograph

## Abstract

We report cases of Armillifer Armillatus infestation in three Nigerian adults within two and half years in our health facility. The first patient was a 70 year old farmer and a regular consumer of snake meat for over 50 years. He presented in February, 2014 for follow-up visit as he was a known systemic hypertensive patient. He was incidentally discovered to have multiple comma-shaped calcific lesions in the lungs and liver on a chest radiograph. These were better demonstrated on abdominal ultrasound and computed tomographic scans. He was asymptomatic. The second patient was a 42 year old male civil servant who presented in December 2015 with dry cough and right loin pain for five and three days respectively. His past medical history revealed that he had been treated previously for pneumonia. He has never eaten snake meat but consumed Alligator (Amphibious reptile) for many years but stopped about 12 years ago. Similar calcific lesions were also noted in his liver and lung parenchyma on chest radiograph and abdominal ultrasound scan. The third patient was an 80 year old man who presented in April, 2014 with dizziness and diminished urine output of one day duration. He was a farmer who has been consuming snake meat for many years, and has been on management for systemic arterial hypertension and prostatic hypertrophy. Chest radiograph and abdomino-pelvic ultrasound incidentally revealed multiple comma-shaped calcific lesions in the lungs and liver. The liver function test parameters were all within normal limits but the electrolytes were deranged and he was anaemic with a Packed Cell Volume of 27%. A diagnosis of Armillifer Armillatus infestation was made in these patients, and they were conservatively managed with Mebendazole. The third case was catherized and the deranged electrolytes were corrected. The first patient was lost to follow-up, whiles the second and third had no remarkable symptoms on subsequent follow-up visits.

## Introduction

The World Health Organisation in its recent estimates of the Global burden of Food borne disease, identified hazards that cause 600 million food borne illnesses and 420,000 mortality [1]. These disease agents include virus, bacteria, aflatoxin and parasites some of which have higher impact on children less than 5 years and persons living in low resource environment with poor hygiene and behavioural practices [1]. The quest for livelihood sustenance and drive for food sources has made other unconventional livestock of interest to low-income individuals in rural Africa. One of such is the habit of eating snakes and other reptiles as protein sources [2]. These reptiles are common in tropical sub Saharan Africa where they co-exist with human and other larger animal in sometime hostile relationship including ability to inflict deadly bites in the bush, farms and sometimes in residential houses [3, 4]. Humans also hunt snake either by roosting them out of bush and/or catch edible ones for meat. Hobby snake keeping and snake farms are found in peri-urban areas in West Africa; this is beside serpents that are kept in research institutions as teaching aids and for the development/production of anti snake venom [5]. Hence the interaction of humans with snakes has increased in recent time with public health consequences. Commonly encountered snakes in Nigeria can be broadly divided into two families: Viparidae which are heamotoxic (carpet vipers (echis carinatus), puff adder), and Elapidae which are neurotoxic (cobras, bungarus) [6]. Some of these snakes are large and meaty and eaters have described the meat as lean, white and palatable [7], which seems to augment sources of animal protein and nutrition in rural Africa. What is however least known is the public health risk associated with snake meat. Also important are pathogens that eaters, handlers and hobbyist may inadvertently be exposed to in their contact with snakes [5]. Armillifer armillatus belong to the order porocephalida in the family Pentastomes. These are group of vermiform snake related parasitic disease that infect humans and animals [5]. The parasites are similar to annelida and arthropoda but are classified as crustacea, probably related to maxillopoda/branchiurans [8]. Armillifer armillatus is predominant in West Africa as a parasite of snakes and other reptiles where they suck blood by means of hooks that anchor them to tissues of the respiratory tract and are responsible for visceral pentastomiasis in humans [9, 10]. On the global perspective, Armillifer armillatus infestation is rare and is of public health importance hence, the prompting of the report.

## Results

### Case presentation and results

**Figure 1:**
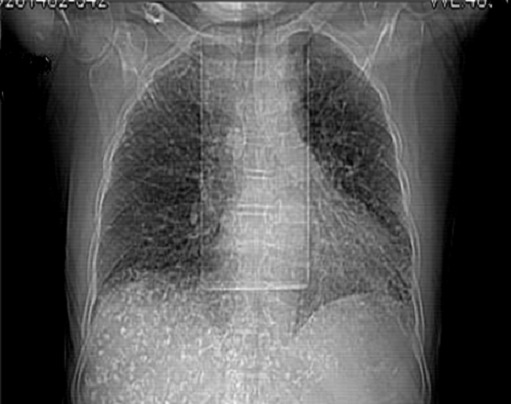
Chest scanogram showing bilateral, multiple, crescentic opacities of calcific density in both lungs and in the region of the liver

**Figure 2:**
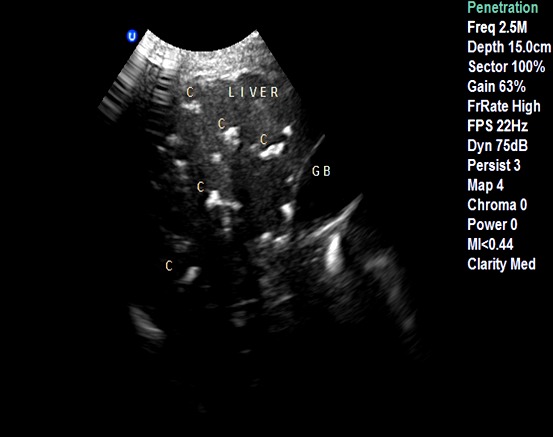
Abdominal ultrasonogram at the level of the liver showing multiple brightly echogenic lesions casting posterior acoustic shadows in the liver parenchyma, distorting its architecture

**Figure 3:**
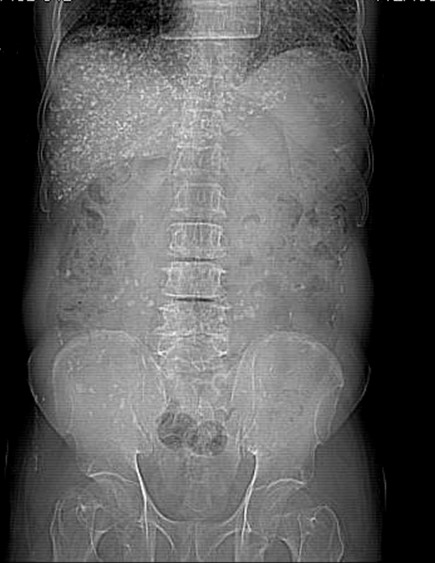
Abdominal CT-scannogram of the same patient showing the numerous comma-shaped calcific densities all over the abdomen, especially over the hepatic region. Degenerative changes are also noted in the lumbar vertebrae

**Figure 4:**
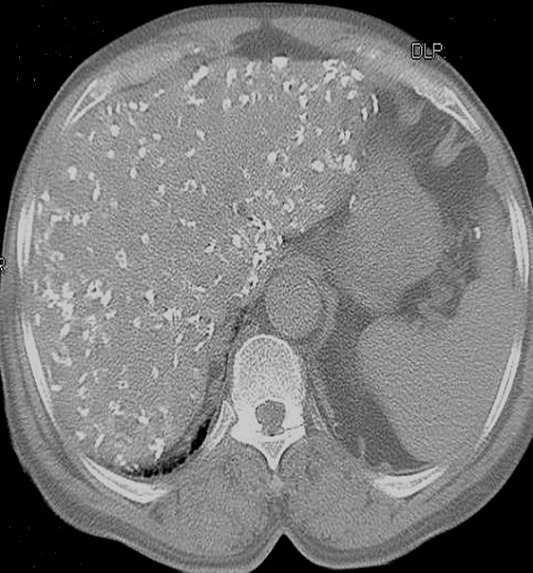
Non-enhanced axial CT-slice of the abdomen at the level of the liver (Bone window) showing multiple hyperdense lesions of calcific density in the liver, with parenchymal distortion; calcific deposits are also noted on the splenic capsule and bowel wall

**Case 1:** A.Y was a 70 year old farmer from North-central Nigeria who presented to our health facility for a routine visit as a systemic arterial hypertension patient. He was asymptomatic at presentation but volunteered a history of prolonged ingestion of snake meat for over 50 years. Systemic examination showed a calm elderly man who was not pale and anicteric. No peripheral stigmata of chronic liver disease were noted in him but there was cardiomegaly with apex beat located at the 6th left inter-costal space mid-axillary line. The heart sounds were normal S1 and S2 only. There was no murmur, and the remaining systems were essentially normal. As part of general assessment of hypertensive patients, he was referred to Radiology department for chest x-ray. This revealed features of hypertensive heart disease evidenced by cardiomegaly of left ventricular preponderance and unfolded aorta. Multiple comma-shaped opacities of calcific density were noted in both lung fields and in the demonstrated segment of the liver shadow (Figure 1). These findings necessitated further investigations such as Abdominal Ultrasound Scan, Computed Tomographic Scan, Liver Function Test, and Full Blood Count and Differentials. The ultrasound scan (Figure 2) showed multiple brightly echogenic oval and comma-shaped lesions casting posterior acoustic shadows in the entire segments of the liver, with few components noted in the splenic parenchyma and the cortices of both kidneys. Thoraco-abdominal computed tomographic scans (Figure 3, Figure 4) gave better characterization of the lesions in the lungs, liver, spleen, kidneys, and peritoneum and in the bowel walls. The liver function test parameters and renal function assessment (urea, creatinine and electrolytes) were within normal limits, and differential white blood cell counts were normal. In view of the aforementioned findings coupled with prolonged history of snake meat consumption, a diagnosis of Armillifer Armillatus infestation with background hypertensive heart disease was made. He was reviewed by a physician and managed conservatively with Mebendazole. He also continued with anti-hypertensive regimen. His response to treatment was good until he was lost to follow-up.

**Figure 5:**
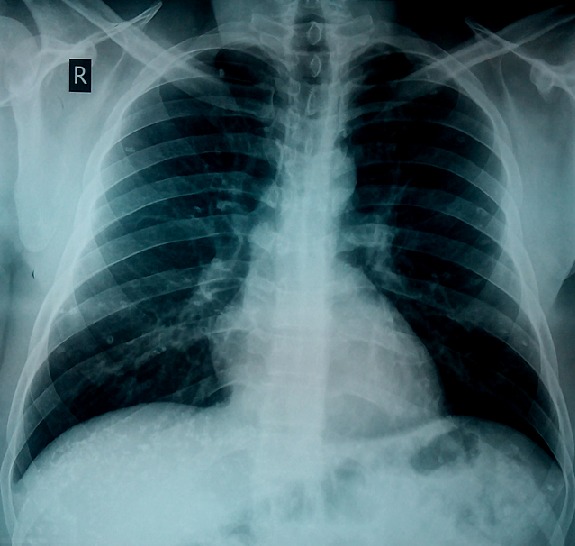
Posteroanterior chest radiograph showing bilateral, multiple, crescentic calcific opacities in both lungs and in the region of the liver

**Figure 6:**
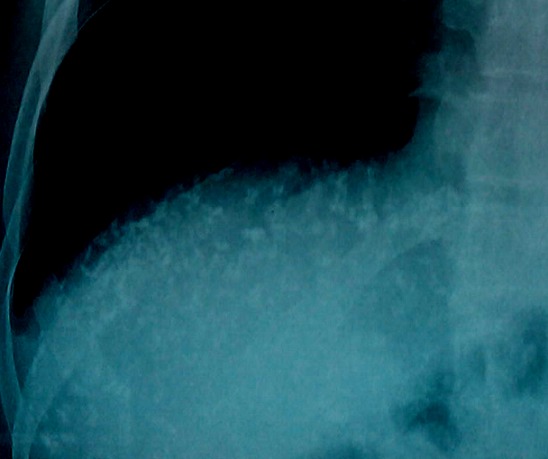
Cone view of the right hypochondrium of case 2 showing multiple crescentic and oval-shaped opacities of calcific density in region of the liver

**Figure 7:**
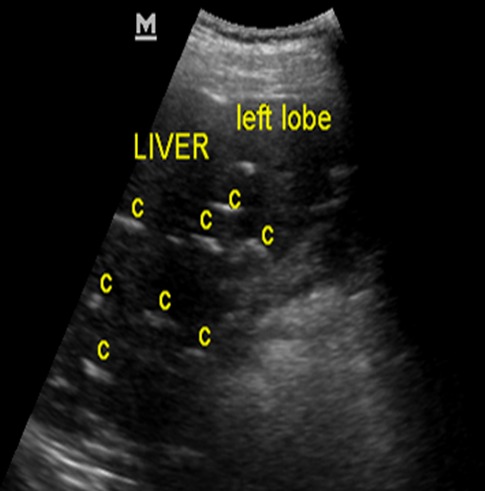
Abdominal ultrasonogram of case 2 at the level of the liver showing multiple brightly echogenic lesions (C) casting posterior acoustic shadows in the liver parenchyma, distorting its architecture

**Case 2:** M.E was a 42 year old civil servant from South-South Nigeria who presented to our health facility in December, 2015 with 5 day history of dry cough and his past medical history revealed that he was treated for respiratory tract infection three months prior to presentation. He also presented with right loin pain of 3 days duration which subsided spontaneously without medication. There was no known aggravating or relieving factors, and no associated dysuria or heamaturia. His bowel habit was not altered, and there was no vomiting or yellowish discolouration of the sclera. He volunteered history of consumption of alligator (Amphibious reptile) for many years, but stopped about 12 years prior to presentation. When examined, he was calm and not in obvious distress. He was clinically stable and systemic examination was unremarkable. Abdominal ultrasound scan and chest x-ray were ordered. The chest X-ray revealed multiple comma-shaped opacities of calcific density in both lungs and in the region of the liver (Figure 5, Figure 6). These were confirmed on ultrasound scan of the abdomen (Figure 7). Widespread brightly echogenic lesions casting posterior acoustic shadows were noted in both lobes of the liver. The remaining abdominal viscera were sonographically preserved. As in the first case, the thoraco-abdominal computed tomographic scan gave better delineation of the aforementioned calcific lesions. In addition, multiple hyperdense lesions of calcific density were noted in the peritoneum and in the bowel walls. A diagnosis of Armillifer Armillatus infestation was made on the basis of the above clinical and radiological findings. He was reviewed by the physician and placed on conservative management (Mebendazole and ciprofloxacin). He is doing well and had no complaints whatsoever on subsequent follow-up visits.

**Case 3:** A.J was an 80 year old man from North-Central Nigeria who presented on the 6th of April, 2014 with a day history of dizziness and diminished urinary output. He was a farmer and has been consuming snake meat regularly for many years. He had also been on management for systemic hypertension and symptoms of prostatism by cardiologist and urologist respectively. The blood pressure at presentation was very low (70/40mmHg). When examined, he was found to be ill-looking, anicteric, acyanosed but mildly pale. His chest was clinically clear. Digital rectal examination showed enlarged, firm and nodular prostate with obliteration of the median groove. He was admitted, catheterized and intravenous line secured while all anti-hypertensive drugs were stopped. Full blood count and differentials, Urea, electrolytes and creatinine, Prostatic Surface Antigen (PSA), Abdomino-pelvic Ultrasound scan, chest X-ray, abdominal Computed Tomography and Liver function test were ordered. Chest x-ray showed features of hypertensive heart disease, while abdomino-pelvic ultrasound scan showed prostatic enlargement (141.6g), urinary bladder calculus and multiple comma-shaped hepatic calcifications in keeping with calcified nymphs of Armillifer armillatus. This was further confirmed on computed tomographic scan. Liver function test parameters were normal, but PSA was elevated (34.04 ng/ml). Urea and creatinine were 13.4mmmol/L and 951.9mmol/L respectively. He was managed conservatively, and on the 6th day of admission, both urea and creatinine became normal. He was later discharged to be followed-up at SOPD and MOPD. On subsequent follow-up, his blood pressure showed marginal systolic hypertension and he was placed on Amlodipine, Frusemide and Lisinopril. As he was asymptomatic in view of the incidental discovery of Armillifer armillatus infestation, he was managed conservatively, while the urologist placed him on Tamsulosin (0.4mg) and Finasteride (5mg) daily in the interim before probable prostatectomy when stable and fit.

## Discussion

Human and animals in a shared environment are constantly exposed to diseases that can be transmitted from one to the other called zoonoses [11]. Infectious diseases are the leading cause of debility and mortality in under developed countries and it has been estimated that greater than 70% of emerging diseases are transmitted directly from animals [12]. The adult parasite of Armillifer armillatus is a degenerate of Arthropod of the family Linguatulidae. Five species have been identified in man, but Porocephalus (Armillifer) armillatus is the commonest, and occurs commonly in central and west Africa. Others are Porocephalus Crotali (North and South America), Porocephalus Subulifer (Africa), Porocephalus Monoliformis and Linguatula Seratta (world wide) [13]. Armillifer armillatus and Armillifer Moniformis are usually responsible for porocephalosis in humans [14]. Adult Armillifer armillatus is found in the trachea and bronchi of pythons, cobra and other reptiles, in Central and West Africa. The adult is vermiform, yellowish and translucent; only the calcified nymphs are visualized radiographically. The eggs which are double-shelled and resistant to water and gastric juice are laid in the posterior portions of the nasal passage of snake and are released from the body in nasal secretions and in feaces. The eggs embryonate upon reaching damp vegetation or water, and are then ingested with contaminated water or food by rodents, monkeys and other animals. Man acquires the infestation by drinking pond water contaminated by snakes or by eating snake meat or any of the aforementioned animals [14].

The first documentation of human infection was by Prunner in Cairo in 1847 [15]. Since then, many cases have been reported in the literature, but it is still relatively rare as only three cases were identified in our centre in about three years. Majority of the cases reported are males, with a male: female ratio of 2:1 [9] which concurs with the observation made in our centre where the three cases seen so far are males. The male preponderance may be due to increased adventure and exposure to infected water via bathing, swimming, and agricultural activities. Most cases of Armillifer armillatus infestations are asymptomatic and few present with mild symptoms as in the second case. This disease is especially seen among those who consume Snake and other reptile's meat, and those that drink water contaminated by snake and infected rodent secretion [14]. The first and third patients admitted history of snake meat consumption while the second has no such history. He however admitted consumption of Alligator. The fact that he is from South-south Nigeria where he may have had several encounter with water contaminated with the parasite coupled with the fact that other reptiles like alligator could be infective may be the reason for his disease [16]. The first and third patients had no features specific for armillifer Armillatus infestation while the second case presented with non-specific dry cough on two occasions at two month interval. Both episodes of cough were treated conservatively and he did well following treatment. The cough may not be related to the infestation, but previous publications by other authors revealed some cases of pneumonitis that occurred as a result of Armillifer armillatus infestations [16]. In patients with severe infestation, there could be associated pericarditis, pleuritis, acute abdominal conditions such as intestinal obstruction and even viscus perforation [17]. The encysted larvae may cause abdominal pain, vomiting, constipation and diarrhoea. These acute abdominal conditions have resulted in unnecessary laparotomy before the true diagnosis was made in some cases and death has been reported among those with severe infestation in some locations [17]. Most of the enumerated conditions were not observed in the three cases other than the mild dry cough that the second patient presented with. The vague right loin pain observed in the second patient may not have direct relationship with the infestation as it subsided spontaneously. Close surveillance using abdominal ultrasound scan, liver function test and faecal occult blood test are advised in view of the radiologically confirmed severe hepatic affectation.

This will go a long way to prevent hepatic failure and even malignant transformation [16, 18]. Armillifer armillatus infestation is usually diagnosed incidentally on radiological assessment of patients for other reasons as observed in the cases presented. Conventional radiographic assessment, computed tomographic scan and ultrasound scan are some of the basic radiological methods available for the assessment of patients with Armillifer armillatus infestation. Our patients were assessed with these modalities and classical radiological findings were noted in all of them. Computed tomograpic scans and conventional radiographs of the first two patients revealed extensive crescentic calcific lesions in the lungs, peritoneum and liver, with highest concentration in the right hypochondrial regions which is consistent with the findings of earlier authors [14, 16]. These radiological features are diagnostic and pathognomonic of Armillifer infestation [14, 16, 19]. Some patients with pentastosomiasis may have deranged liver function test parameters. This is usually seen in those with severe liver affectation as noted by some authors [16]. Our patients however had normal liver function test parameters despite severe liver involvement. This may be due to high residual functional capability of the liver as documented by other authors [20]. Liver biopsy is required as complementary investigation tool necessary to diagnose Pentastosomiasis [16, 19], but our patients did not consent to it.

The treatment of Armillifer armillatus infestation is usually conservative, as the majority of the cases are asymptomatic [16, 19]. Few patients that are symptomatic may require surgical intervention, especially those that present with intestinal obstruction or perforation [19]. Since our patients were not symptomatic, let alone acute abdominal condition that could warrant surgical intervention, there was no need for surgical management in any of them. No established anti-parasitic chemotherapy is available for pentastomiasis; however, Mebendazole has been suggested for those with mild to moderate infestation [19]. Our patients were treated with Mebendazole, and the second patient also had a full course of ciprofloxacin for the cough he presented with. The first patient defaulted from subsequent follow-up, while others are being followed-up closely at the medical out-patient and surgical out-patient departments of our institution. As at their last visits, none had significant complaints.

## Conclusion

These cases are presented to bring to the notice of the public, Radiologists and other Clinicians the pathognomonic radiological findings and the possible clinical relevance of Armillifer armillatus infestation following consumption of snake meat and/or other reptiles such as Alligator. The public health hazards associated with reptile's meat, particularly snake and contaminated water sources have also been stressed.

### What is known about this topic

- Armillifer Armillatus is known to occur among those that consume snake meat;
- Severe liver affectation often results in deranged liver function test (LFT) parameters, and even malignant transformation in some cases;
- Most Armillifer Armillatus infections are asymptomatic but fatal consequences such as intestinal obstruction, viscus perforation, malignant transformation and hepatic encephalopathy have been reported in few cases.

### What this study adds

- Alligator (Amphibious reptiles) is now known to be one of the reptiles that harbour Armillifer Armillatus ova and human that consume this meat could also be infected with the disease. The second patient consumed Alligator, and never snake meat;
- Despite severe liver affectation by the calcified nymphs of Armillifer Armillatus in most of the patients presented, the liver function test parameters of the patients were all within normal limits. This may be due to high residual functional capability of the liver;
- Farmers were known to be affected in most of the previous publications, but a civil servant (one of the cases presented) was infected. High index of suspicion is therefore imperative in all cases, irrespective of age or occupation of the patients being evaluated.
